# Sector-differences in Adults’ Dental Care Service Utilisation: 11-year Register-based Observations

**DOI:** 10.1016/j.identj.2024.12.035

**Published:** 2025-02-06

**Authors:** Miira M. Vehkalahti, Ulla Palotie, Sinikka Varsio, Kaija Hiltunen

**Affiliations:** a Department of Oral and Maxillofacial Diseases, University of Helsinki, Helsinki, Finland; b Oral Diseases Teaching and Dental Care Unit, Helsinki University Hospital, Helsinki, Finland; c Social Services and Health Care Sector, Helsinki, Finland

**Keywords:** Adults, Dental services, Overtreatment, Private sector, Public sector, Undertreatment

## Abstract

**Objective:**

This register-based study evaluated sector-specific differences in adults’ utilisation of dental care services in Helsinki, Finland in 2007-2017.

**Methods:**

The target population comprised all inhabitants aged 20+ years in Helsinki over an 11-year period, from 2007 to 2017. The data, aggregated into 5-year age groups by treatment year and type of treatment, included inhabitants with at least 1 visit to a dentist in the private or public sector. In 2007-2017, the numbers of all patients ranged between 229,772 and 261,488. The patient age groups were analysed for periodontal, restorative, endodontic, and oral surgery treatment received. Attendance rates (%) refer to the number of patients per number of inhabitants. Proportions (%) of patients receiving various treatment types refer to numbers of patients per all patients. Comparisons between the age groups included percentual change in absolute numbers and percentage points in rates. The treatment-year trends were analysed by applying linear regression models.

**Results:**

Attendance rate for all adult patients was 49.5% in 2007 and 48.8% in 2017. During the 11-year period, adults’ attendance to private service decreased (31.4%-24.9%) but increased to public service (18.1%-23.9%). Patients receiving periodontal care increased in both the private (67%-72%) and public (36%-45%) sectors, while patients with restorative care decreased in both sectors private (67%-58%) and public (58%-48%). Further, fewer private- than public-sector patients received oral surgery or endodontic treatment.

**Conclusions:**

Sector-specific differences in patient treatments may indicate over- or undertreatment choices in restorative and periodontal care.

**Clinical Relevance**

The sector-specific differences found could arise from uncertainties which complicate clinical decision making in both sectors but also encourage private and public dentists to improve the quality of clinical decision-making to attain the preselected goals of patient's health, well-being, and satisfaction. Bearing in mind the benefit of the patient, avoiding over- and undertreatment is feasible despite the sector.

Alt-text: Unlabelled box

## Introduction

Wide variation in dental care service provision in European countries is apparent according to reports from Chief Dental Officers.^1^ The Nordic countries are similar in that they provide subsidised public and private services for all adults. In Finland, 40% of dentate adults reported the use of private and 21% public dental care services in 2000.^2^ In 2002, a reform stipulated subsidised dental care for all citizens, and soon a shift was noted from private to public services, with 37% of patients reporting use of private and 29% public dental services in 2007,^3^ and in 2011, 34% and 24%, respectively.^4^

In other Nordic countries, organisation for administration and financial aspects of dental care services varies from country to country, and the service usage figures exceed the figures in Finland. In Sweden, a follow-up of a 50-year-old age cohort in 2 counties stated annual use of dental services for 92.3% in 1992 and for 87.2% in 2007,^5^ while in 2013 a telephone interview disclosed regular dental visits for around 90% of respondents in a nationwide random sample of 3500 adults.^6^ In Denmark, a recent study summarised nationwide representative data, obtained from surveys conducted in 7 waves during 1987-2017, and showed an increase in adults’ regular dental visits, from 66.3% to 72.1%.^7^ In Norway, for randomly selected noninstitutionalised adults from the cross-sectional health survey in 1975-2018, a notable increase in rates of dental visits was found: from 59.2% to 80.4%.^8^

In 2009, in the United Kingdom, 73% of dentate adults reported a visit to a dentist in the previous 12 months and 45% of users reported using paid National Health Service (NHS) dental care and 25% free NHS dental care, while 27% reported using private dental care services.^9^ In Germany, a study based on evidence of the actual use of services with various types of insurance data reported that the annual rate for utilisation of dental services was around 70% in 2010-2018.^10^ In the United States, 40.5% of adults visited a general dentist (GD) in 2001 and 37.0% in 2010, based on the Medical Expenditure Panel Survey (MEPS) data and including GD providers only.^11^ In Germany and in the United States, adults’ dental care services are mainly provided by private-sector dentists.^10^^,^^12^

Restorative and periodontal care tend to predominate in service provision regardless of the type of research data. In Finland, 66% of the public sector adult patients reported having received a ‘filling’ and 61% ‘polishing or scaling’, while the corresponding figures for the private sector were 64% and 75%, respectively.^2^ A register-based study of dental care for adults in Finland in 2009 revealed that periodontal and restorative treatment measures were more frequent in the private sector, while in the public sector oral surgical measures were more frequent.^13^ Later, register-based studies of private sector dental care report that in 2017 altogether 61.1% of patients received restorative treatment,^14^ 13.8% oral surgery,^15^ and 8.9% endodontic treatment.^16^ For other countries, no sector-based reports were found.

We assumed that adult's shift from private to public sector will not lead to considerable differences in usage and selection of treatments. The aim of this register-based study was to evaluate sector-specific differences in the use of dental care services by the adult population in Helsinki, Finland, in 2007-2017.

## Methods

### Background

In Finland, dental care services for adults are provided in both the private and public sectors, and adults can freely choose between the sectors. All inhabitants are insured by the Social Insurance Institution of Finland (SII) and will receive partial subsidisation of the costs paid for private sector services, excluding orthodontic and prosthetic treatments and aesthetic procedures. Public sector services are organised by each community for its inhabitants. The services are highly subsidised, and the fees that adults pay are clearly below those charged in the private sector where a dentist can define the charged fee for each procedure. In both sectors, dentists record the details of the treatment measures using the same codes maintained by SII for electronic recordings of treatments given. Accordingly, the fees are determined by SII codes both for subsidisation in the private sector and, in the public sector, for administrative defining patient charges and incentives to salaried dentists. Despite different providing schemes in both sectors, the registered treatment data cover all procedures for all patients and are similarly coded. The SII creates an open database of the numbers and types of treatments and aggregates the data according to the residency and age of private sector patients by calendar year. Within each community, the public service administration produces similar numerical data by calendar year, but the use of the data is subject to administrative permission.

### Data collection and definitions

For this study, the target population comprised all inhabitants aged 20 years and over in the City of Helsinki over an 11-year period, from 2007 to 2017, separately for each calendar year. For each year, the data included the service information all over across the year, from January 1 to December 31. Data of the population aged 20 years and over were extracted from official statistics and were already aggregated into 5-year age groups from 20 to 24 years onwards.^17^

Those inhabitants who had at least 1 visit to a dentist in the private or public sector were included as patients of that year.

Before delivering the register-based data of dental care services to our use, all information on the numbers of patients in total and by treatment type was aggregated according to each sector by treatment year. Further, detailed data for 3 years (2007, 2012, and 2017) were given for 5-year patient-age groups, from 20-24 years to 99+ years. For the analyses, we combined the oldest groups into one, those aged 90 years and over.

A patient was categorised into subgroups by treatment type to cover all treatments he/she received and could thus belong to several subgroups. The specification of treatment types followed the SII codes, which were the same in both sectors. The patient subgroups selected here for analyses covered periodontal, restorative, endodontic, and oral surgery (mainly tooth extractions) treatment recipients. Starting in 2010, the registration of periodontal treatment procedures expanded when oral hygienist care was included in the subsidisation scheme as part of dental care. The 4 main categories were selected to allow comparisons with other countries. This study analysed as observation elements the subgroups of patients, and thus, had no opportunity to evaluate individual features.

### Ethical considerations

Permission to use the data came from the administration of the SII and Helsinki City, both of which provided us with aggregated data based on official records. Since all data were register based and already aggregated, no identification for individual patients was available and no ethics permit was needed.

### Study population

In the City of Helsinki, the population aged 20 years and over ranged from about 464,000 in 2007 to about 536,000 in 2017.^17^ In these target populations, the number of studied patients ranged by year from 229,772 in 2007 to 261,488 in 2017. Only the patient's age (age group), no other sociodemographic information, such as sex or socioeconomic status, could be included in the aggregated data provided by the SII and Helsinki City administration.

### Data analyses

We outlined indicators of the use of services separately for each age group and calendar year. Attendance rates (%) refer to the number of patients per number of Helsinki inhabitants. Proportions (%) of treated patients in the selected categories refer to numbers of such patients per all patients in the observation period. Volume of each treatment type was defined separately for both sectors as proportion (%) of such patients among all patients having used dental care services. The rates and proportions were calculated per calendar year and separately for private and public services. Curves describing the proportions of patients receiving treatments in the main categories were shown in 2 illustrations using scales suitable for the number of users in each service type. We assessed the changes in attendance from 2007 to 2017 as a percentual change in absolute numbers and as percentage points, ie, differences between the rates. For each year, differences in the attendance rates between the service sectors were shown as percentage points (pp). Findings in the selected 3 years, ie, 2007, 2012, and 2017, allow comparison within the age cohorts, as, eg, those aged 20 to 24 years in 2007, who were 25 to 29 years in 2012, and 30 to 34 years in 2017. We calculated age-standardised attendance rates for the 3 years, overall and by sector, using weights by the population data of the citizens aged 20 years and over.

We evaluated the role of time (calendar year) in the trends of dental care by means of linear regression modelling. Its results provide regression coefficients (β) to describe trend slopes. Analyses and graphical illustrations were performed with Survo MM software (version 3.4.1; Survo Systems, Helsinki, Finland).

## Results

The number of patients studied aged 20 years and over ranged from 229,772 in 2007 to 261,488 in 2017, denoting the range in overall attendance rates from 49.5% in 2007 to 48.8% in 2017 (Table 1). The attendance rate for private services ranged from 31.4% in 2007 to 24.9% in 2017 and for public services from 18.1% to 23.9%, respectively. Table 1 shows further details of population, patients, and attendance rates by calendar year. Across the 11 years, the overall attendance rate stayed around 50%, while the difference between sectors decreased from 13.3 pp in 2007 to 1.0 pp in 2017. Within the private sector, the 11-year change was -6.5 pp and within the public sector +5.8 pp.

**Table 1:** Numbers of individuals aged 20 years and over in the population and numbers of patients utilising dental care services in Helsinki, Finland, in 2007–2017.

Year	Population	No. of patients			Attendance rate*			
	All	All	Private	Public	All	Private	Public	Sector Δ
	×1000	×1000	×1000	×1000	%	%	%	pp
2007	464	230	146	84	49.5	31.4	18.1	13.3
2008	468	239	145	94	51.1	31.0	20.1	10.9
2009	475	247	146	101	51.9	30.7	21.2	9.5
2010	483	244	141	103	50.5	29.2	21.3	7.9
2011	490	250	150	100	51.1	30.7	20.4	10.3
2012	497	256	152	104	51.4	30.5	20.9	9.6
2013	505	263	155	108	52.2	30.8	21.4	9.4
2014	513	262	151	111	51.1	29.5	21.6	7.9
2015	518	277	151	126	53.4	29.1	24.3	4.8
2016	524	266	139	127	50.8	26.5	24.3	2.2
2017	536	261	133	128	48.8	24.9	23.9	1.0
11-year Δ	+72	+31	–12	+44	–0.7 pp	–6.5 pp	+5.8 pp	–12.3

⁎ Attendance rates shown for all and by service sector. Sector Δ = difference from private to public sector as pp; pp = percentage point; 11-year Δ = difference from 2007 to 2017 as counts and pp.

**Fig. 1:**
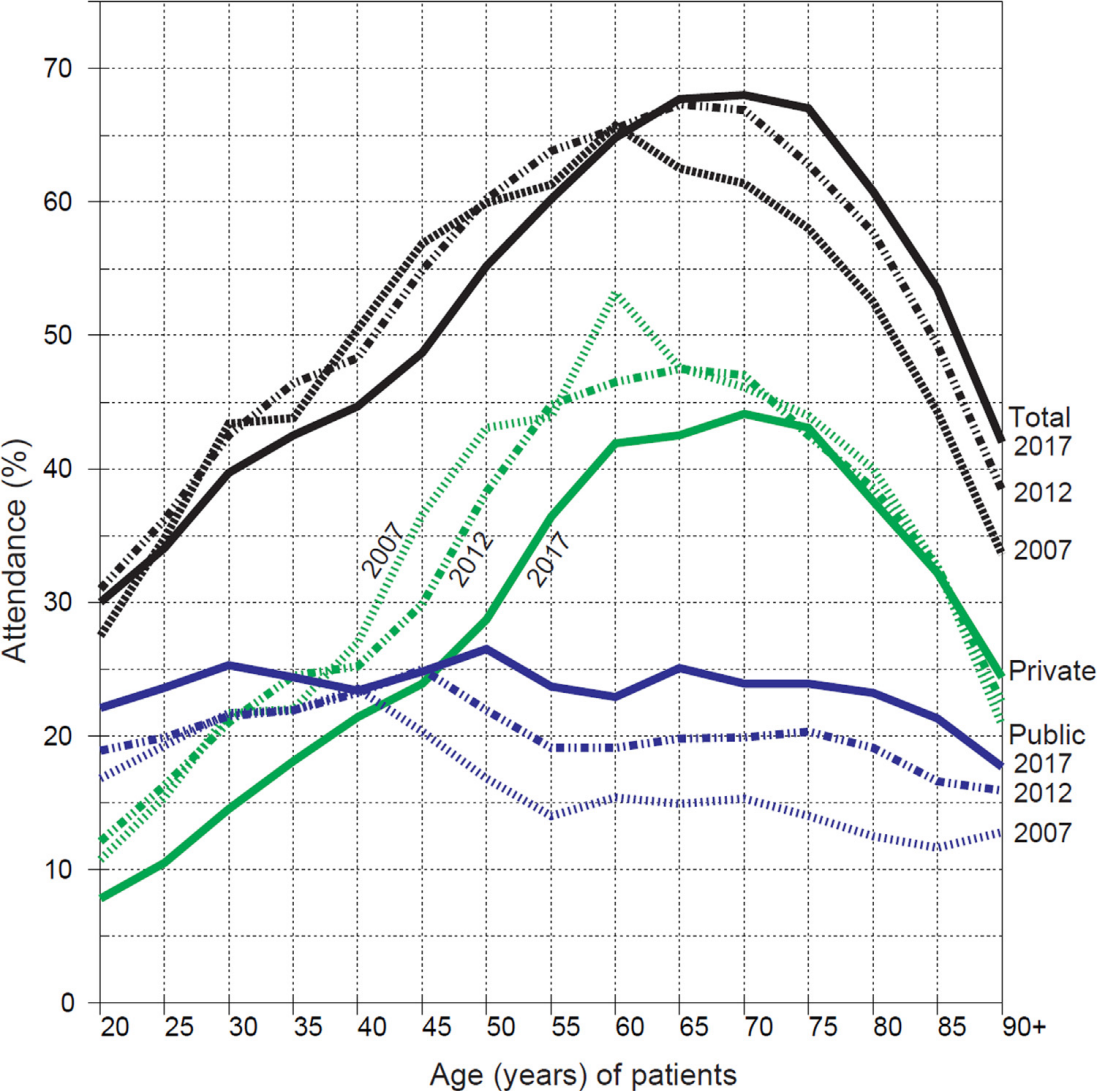
Attendance (%) to dental care by age among citizens aged 20 years and over in Helsinki, Finland, in 2007, 2012, and 2017. Curves for overall attendance are in black, for the private sector in green, and for the public sector in blue.

Attendance rates varied by age group, the smallest rates being for the youngest and the oldest age groups. Figure 1 shows attendance rates by age group in 3 selected years for all patients and separately by service sector. Age-standardised overall attendance rates were 49.5% in 2007, 51.1% in 2012, and 49.0% in 2017. The corresponding figures for private sector were 31.4%, 30.3%, and 25.0% and for public sector, 18.1%, 20.8%, and 24.0%. For each year, the shapes for all patients were relatively similar, showing, however, an increase in attendance in 2017 for those aged 65 years and older. Differences by age were notable in the private sector, where treatment of patients aged 45 to 80 years dominated. Compared to private sector, attendance rates for public sector services showed considerably smaller differences by age group. Comparison between same-age groups in the 3 years, vertically seen in the graph, reveals a clear decrease in attendance to private services up to the age of 75 years, whereas a clear increase in attendance to public services is seen for the age groups of 50 to 54 years and onwards. Horizontal comparison exposes changes within age cohort groups (Figure 1).

**Fig. 2:**
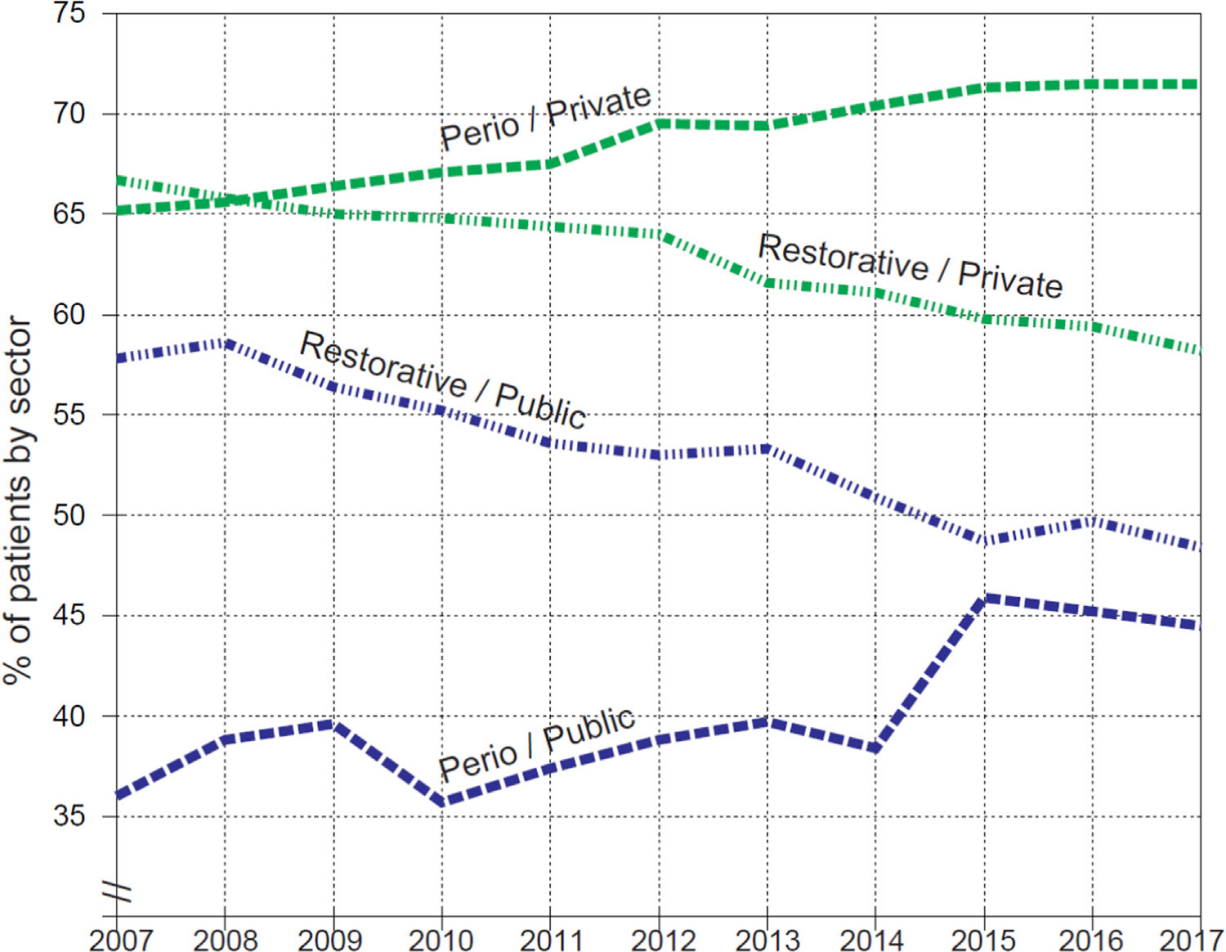
Proportions (%) of patients aged 20 years and over receiving restorative and periodontal care in the private and public sectors in Helsinki, Finland, in 2007–2017.

The most received treatment types were periodontal and restorative care, both types being more frequently received by patients visiting a private-sector dentist (Figure 2). In 2007, 65% of private sector patients received periodontal care and 67% restorative care. In the public sector services, the corresponding figures were 36% and 58%. In both sectors, the proportions of patients receiving periodontal care increased across the 11 years, in 2017 being 72% in private services and 45% in public services. The corresponding proportions of restorative care patients decreased to 58% in private services and to 48% in public services. Considerable annual sector differences occurred in the proportions of periodontal care recipients with a range for private sector (65.2%-71.5%) and for public sector (35.7%-45.9%), proving a 25 to 32 pp difference by year. Correspondingly, the figures for proportions of restorative care, all in decrease, ranged for private sector (66.7%-58.2%) and for public sector (58.6%-48.4%), proving a 7 to 11 pp difference by year.

**Fig. 3:**
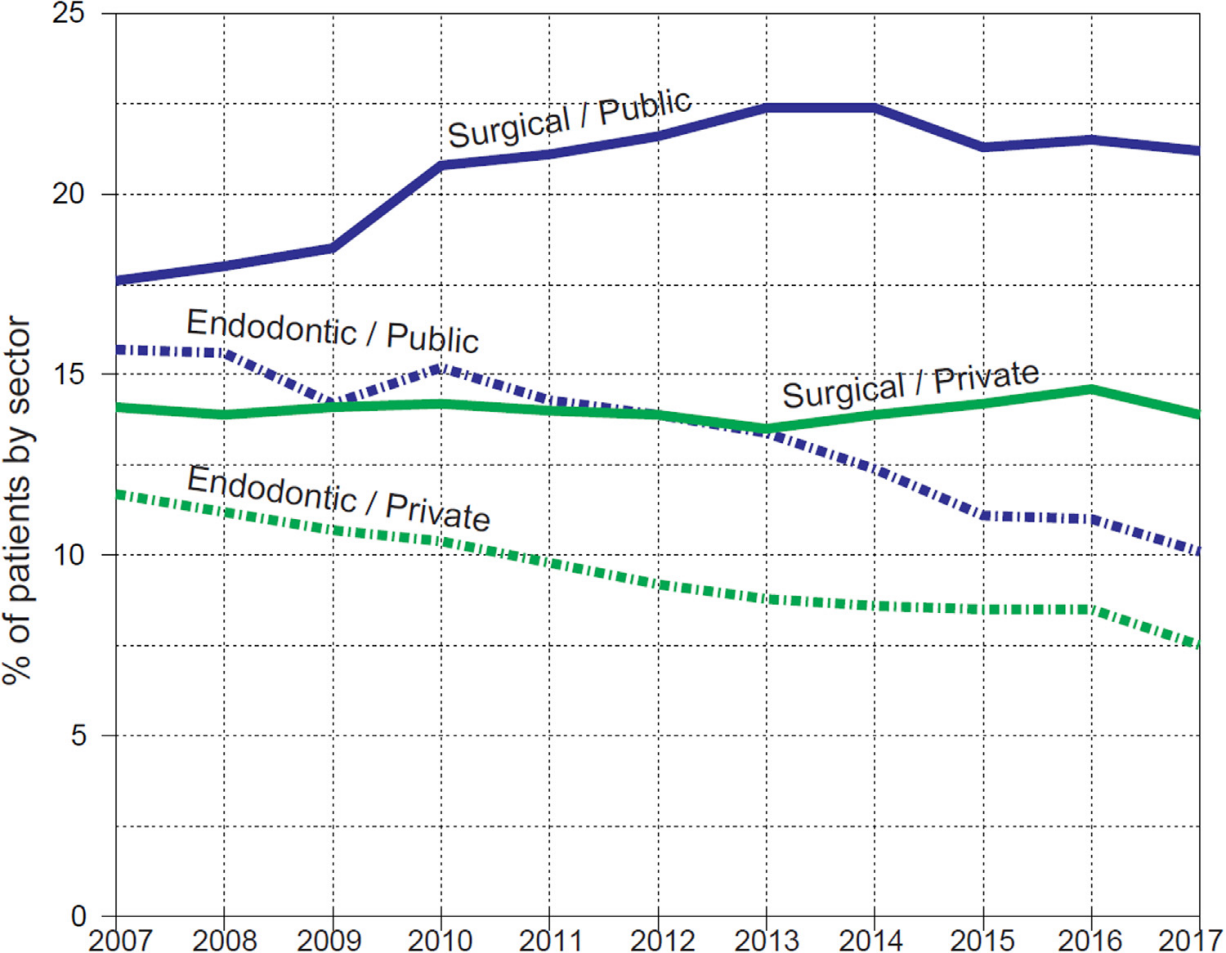
Proportions (%) of patients aged 20 years and over receiving endodontic and oral surgical care in the private and public sectors in Helsinki, Finland, in 2007–2017.

Oral surgical care (mainly tooth extractions) and endodontic care were less frequently received in private services than in public services (Figure 3). In 2007, 14% of private sector patients received surgical care, and the rate was the same in 2017. In public services, the proportions of surgical care patients increased from 18% in 2007 to 21% in 2017. The proportions of endodontic care patients decreased in both service sectors, in private services from 12% to 8% and in public services from 16% to 10%.

Trends in attendance and changes in the volume of treatment are shown in Table 2. Linear regression models revealed how the year of treatment explained changes in attendance to services and in the number of patients receiving each category of treatment. Trends were consistently on the rise for both sectors in provision of periodontal care, while declining in provision of restorative and endodontic care. Opposing trends were obvious for attendance to services, as the public sector had an upwards trend and the private sector a downwards trend.

**Table 2:** Attendance to and changes in the volume of dental care in Helsinki, Finland, in 2007–2017, explained by year of treatment using regression modelling separately for private and public services.

Attendance rate by type of care and provider	Regression coefficient (95% confidence interval)*	Standard deviation	t	*P*
Attendance (any care)				
Private	–0.501 (–0.535, –0.467)	0.122	–4.106	<.01
Public	0.515 (0.491, 0.539)	0.086	5.982	<.001
Periodontal care				
Private	0.708 (0.695, 0.721)	0.046	15.54	<.001
Public	0.859 (0.798, 0.920)	0.221	3.890	<.01
Restorative care				
Private	–0.854 (–0.869, –0.839)	0.054	–15.77	<.001
Public	–1.040 (–1.06, –1.02)	0.078	–13.29	<.001
Oral surgical care				
Private	0.002 (–0.005, 0.009)	0.027	0.090	ns
Public	0.410 (0.381, 0.439)	0.105	3.896	<.01
Endodontic care				
Private	–0.392 (–0.399, –0.385)	0.025	–16.00	<.001
Public	–0.564 (–0.579, –0.549)	0.054	–10.38	<.001

⁎ Positive regression coefficients indicate an upwards trend and negative regression coefficients a downwards trend. ns, not significant.

## Discussion

### Main findings

Sector-specific differences were obvious in various perspectives and against our assumption. The age-based difference in attendance to private services was notable and attendance rate curves across the 11-year period had a similar form, while the outline for attendance rates within public services by age group was steady and showed rather similar outlines with a considerable increase by year of treatment. Higher attendance rates for middle-aged groups in private than public sector may indicate patient's loyalty to a long-standing patient-dentist relationship, while younger age group patients may be more familiar with varying dentists in the public service system. In our aggregated data, population demographics and well-being indicators in the City of Helsinki were not available for dental service user groups by sector and could thus give no explanation for the sector differences found. Besides, private and public healthcare activities are based on a different logic as the public sector is responsible for health services for the entire population, while the private sector can choose its patients.

In both sectors, surprising findings across the 11 years were the growth of the proportion of patients receiving periodontal care and, in the private sector, the notable dominance of periodontal care. The Finnish Current Care Guideline^18^ for periodontal care was first published in 2010, updated in 2016 and in 2019, and may thus have increased emphasis on periodontal care and improve dentists’ knowledge of the harmfulness of the most common oral diseases to general health. We also found obvious differences in periodontal and restorative care between the sectors, in favour of the private sector. Further, the findings confirmed sector-specific differences in tooth extractions and endodontic care, both being more frequent in the public sector and probably due to the sector's tight responsibility to provide acute care.

For the private sector, the curves showing the profusion of periodontal treatment received seem to indicate realisation of the actual needs for periodontal care. Moreover, the findings may indicate more accurate diagnosis and implementation of periodontal care in the private sector, while in the public sector, periodontal problems appear to be slightly underestimated and consequently neglected. A register-based study in Finland showed a notable increase in numbers of private sector periodontal patients since 2010, as a result of including the treatments carried out by oral hygienists in the registered data of subsidised dental care.^19^

In 2000, a representative nationwide clinical survey of adults in Finland found that periodontitis (at least 1 deepened pocket exceeding 4 mm in depth) occurred in 64% of all patients, 71% of men, and 57% of women, and in 2011, in 70% of men and 58% of women.^20^ Further, in 2000, the paper reports a need for restorative therapy in 41% of men and 28% of women, and in 2011, in 44% of men and 25% of women. Contrary to these findings, our register-based study demonstrates for both sectors considerable overflow in restorative care. Moreover, it may indicate that about half of the patients received replacement of fillings, probably due to loss, fracture, or poor form of restorations, ranked as the most frequent reasons in a questionnaire among the dentists in Finland.^21^ In everyday life, the acute dental care services, available on a daily basis in the public sector, often take care of such events and record all procedures as ruled.

On the other hand, the lesser of endodontic treatments and tooth extractions could speak for reduced severity of diseases in patients in the private sector, or just be a consequence of public sector acute care responsibilities. Still, these interpretations might be misleading. In line with previous papers from Ireland,^22^^,^^23^ our findings raise questions about the validity of register-based information on dental care to predict treatment needs and to describe dental health in the patient population. Instead, treatment panorama and selection of procedures may describe a dentist's way of reaching preferred goals: patient's health, well-being, and satisfaction with the dental care received, and from the dentist's end: satisfaction with one's educational and professional competence, economic status, and overall progression.

For patients, money and service pricing may be influential in choices between private and public dental care. Studies from 2 cities in Finland report how in Turku ‘private practitioners systematically classified the treatment procedures they provided as more demanding, and therefore more economically rewarding, than their public sector counterparts’^24^ while the Oulu paper revealed ‘a clear socioeconomic gradient for the probability of visits according to income and education: the higher the income and the higher the education, the more likely was a visit to a dentist – especially a private dentist’.^25^ In our study, population demographics was uniform and could thus make no difference between the sectors even though the positive development figures across the 11-year period showed continuous growth of the population and notable increase in the proportion of tertiary education, from 35.2% to 42.2%.^17^

Further, in the Turku study, dentists assessed the demandingness of the treatments, and private dentists, less often than public dentists, assessed the procedure as simple or less demanding: the differences were considerable and were reflected in the higher costs of the private sector.^24^ A large health economic study on the use of dental X-ray as diagnostic tool in the General Dental Service in Scotland stated that ‘there are significant increases in X-rays when dentist receive fee-for-service rather than salary payments and when patients are made exempt from payment’.^26^ An interview among general dental practitioners noted a need to provide more clarity in shared decision-making in person-centred dental care.^27^ A recent questionnaire survey in Norway revealed that patient shortage and fall in demand for the services led private dentists to suggest more treatments and raise the fees to reach and maintain their own income level.^28^ In Finland, however, decision about the treatment methods are made by both the patient and the dentist as the law on the patient's status and rights requires doing so.^29^

In the private sector, the patient's requirement to receive top-level care, such as proper management of pain, fear and anxiety, a sufficient description of the planned care, a well-functioning outcome as well as adequately acceptable appearance, may explain why the dentist suggests choices that might finally lead to overtreatment. Regardless of the situation, the patient's needs and expectations should be heard and the patient's active role in the treatment planning strengthened, thus keeping the patient involved in the treatment process.^30^^,^^31^ In the private sector, dentists may realise patient expectations by producing perhaps too much dental care. By contrast, in the public sector, the patient's requirements for desired treatment probably receive less positive reaction due to patient overload and potential monitoring of the dentist's treatment decisions. In the near future, value-based healthcare may, however, get a more important role in patient-focused, well-prioritised treatment.^32^

### Strengths and limitations of the study

The main strength of the study is that it comprises register-based data for the entire target population. Such data hinder any skewness caused by missing cases and systematic errors in data collection. Further, both sectors have the same SII codes for recording the treatment procedures, and consequently, the reliability of the findings can be deemed convincing.

Limitations are due to the use of aggregated data that mask individual sociodemographic characteristics of patients other than age, which is given as 5-year groupings. Furthermore, the oral hygienist treatment records were not included in the official SII statistics before the year 2010. As expected, after the expansion the data showed a notable growth in the number of periodontal treatments. In addition, no data of dentists’ background characteristics are available. Consequently, our study found no reason for the sector difference, calling for further research to clarify the role of patients’ and dentists’ individual characteristics in selecting treatments.

### Comparison with previous research findings

The overall annual attendance at dental care services in Helsinki was around 50%. This is clearly below the rates reported for services in other Nordic and European countries but near the figures from a nationwide survey in Finland. In 2000, 52% of adults reported utilising dental care services in the past 12 months and a further 17% in the past 1 to 2 years,^2^ while in 2011, 57% reported visiting a dentist during the past year.^4^ A questionnaire among chief dentists confirmed the 2008 attendance rate of 51.6% for the working-age population and 40.9% for those aged 65 years and over.^33^ According to our results, it can be assumed that almost all citizens used the dental care services, but not annually. Consequently, it is hardly surprising that periodontal and restorative care played such a large role in dental services in Helsinki.

Unfortunately, the recording scheme varies from country to country, hampering comparisons between the main types of services from different countries. Proportions of periodontal care patients range from 50% in the United Kingdom^9^ to 26.9% in Germany^10^ and 1.8% in the United States,^34^ while our study in Helsinki found the proportions from 2007 to 2017 to range between 65% and 72% in the private sector and between 36% and 45% in the public sector. The figures for restorative dental care patients were from 28% in the United Kingdom^9^ to 26.5% in Germany^10^ and 19.5% in the United States,^34^ while in Helsinki the ranges by year were 66% to 58% in the private sector and 58% to 49% in the public sector. Proportions of oral surgery patients were 14% in the United Kingdom,^9^ 8.3% in Germany,^10^ and 9.7% in the United States,^34^ while our figures by year were around 14% in the private sector and 18% to 21% in the public sector. It is unlikely that the wide variation in the use of dental care in the countries reviewed here could indicate real differences in the oral health of the population. In contrast, further studies directed to financial aspects could open new paths to understand and find sound reasons for actual sector differences in dental care services.

## Conclusions

Sector-specific differences in patients’ treatments may indicate over- or undertreatment choices, especially regarding restorative and periodontal care. Further, in the public sector the growing congestion of patients may lead to misjudgements of patients’ actual needs and lack of comprehensive care.

## CRediT authorship contribution statement

**Miira M. Vehkalahti:** Conceptualization, Data curation, Methodology, Visualization, Writing – original draft. **Ulla Palotie:** Conceptualization, Methodology, Writing – review & editing. **Sinikka Varsio:** Conceptualization, Data curation, Methodology, Writing – review & editing. **Kaija Hiltunen:** Conceptualization, Data curation, Methodology, Writing – review & editing.

## Conflict of interest

The authors have no conflict of interest.
